# Roles of Hcp2, a Hallmark of T6SS2 in Motility, Adhesive Capacity, and Pathogenicity of *Vibrio alginolyticus*

**DOI:** 10.3390/microorganisms11122893

**Published:** 2023-11-30

**Authors:** Shuilong Wu, Jufen Tang, Bei Wang, Jia Cai, Jichang Jian

**Affiliations:** 1 Guangdong Provincial Key Laboratory of Aquatic Animal Disease Control and Healthy Culture, College of Fishery, Guangdong Ocean University, Zhanjiang 524088, China; 2 Key Laboratory of Diseases Controlling for Aquatic Economic Animals of Guangdong Higher Education Institutions, College of Fishery, Guangdong Ocean University, Zhanjiang 524088, China; 3 Central People’s Hospital of Zhanjiang, Zhanjiang 524045, China

**Keywords:** *hcp2*, T6SS, motility, adhesion, pathogenicity, zebrafish, *Vibrio alginolyticus*

## Abstract

The type VI secretion system (T6SS) is a large secretory device, widely found in Gram-negative bacteria, which plays important roles in virulence, bacterial competition, and environmental adaptation. *Vibrio alginolyticus* (*V. alginolyticus*) is an opportunistic pathogen that causes vibriosis in aquaculture animals. *V. alginolyticus* possesses two type VI secretion systems (named the T6SS1 and T6SS2), but their functions remain largely unclear. In this paper, the roles of the core component of the T6SS2 cluster of *V. alginolyticus* HY9901, hemolysin-coregulated protein2 coding gene *hcp2*, are reported. Deletion of *hcp2* clearly impaired the swarming motility, adhesive capacity, and pathogenicity of *V. alginolyticus* against zebrafish. Furthermore, transmission electron microscopy (TEM) found that the abnormal morphology of flagellum filament in the *hcp2* mutant strain could be partially restored by *hcp2* complementarity. By proteomic and RT-qPCR analysis, we confirmed that the expression levels of flagellar flagellin and assembly-associated proteins were remarkably decreased in an *hcp2* mutant strain, compared with the wild-type strain, and could be partially restored with a supply of *hcp2*. Accordingly, *hcp2* had a positive influence on the transcription of flagellar regulons *rpoN*, *rpoS*, and *fliA*; this was verified by RT-qPCR. Taken together, these results suggested that *hcp2* was involved in mediating the motility, adhesion, and pathogenicity of *Vibrio alginolyticus* through positively impacting its flagellar system.

## 1. Introduction

The type VI secretion system (T6SS), an inverted bacteriophage-like complex that was first defined in *Vibrio cholerae* [1], plays key roles in the infection of eukaryotic cells [2] and bacterial competition [3,4]. *V. alginolyticus* has two T6SS gene clusters (T6SS1 and T6SS2) [5] and encodes homologs of 13 characteristic core proteins [6,7]. One of these components, hemolysin coregulated protein (Hcp), is the hallmark of the T6SS and serves as a key effector for bacterial pathogenicity [8,9,10]. Although Hcp proteins share a highly similar structure in various bacteria, the functions of Hcp proteins are distinct. Recently, it is reported that Hcp is involved in mediating T6SS biofilm formation, bacterial adhesion, virulent regulation, environmental stress adaption, etc. [11,12,13]. More importantly, there are reports showing that the deletion of the T6SS genomic island (GI) or its other core components, such as Hcp, will result in less-motile, lower-virulence bacteria than wild-type strains by regulating the flagellar system at the levels of transcription and translation in several species [14,15,16,17]. 

Bacterial motility relies on the polar flagellum, which is composed of three major structural components: the basal body, the hook, and the filament [18]. Polar flagellar biosynthesis and assembly is a tightly transcriptional hierarchy process regulated by four promoters, named Class I~IV in the *Vibrio* species [19]. During flagellar assembly, flagellar gene expression is controlled by several master regulons. RpoN (σ54), a type of transcriptional regulatory factor, is widely identified in pathogenic bacteria. RpoN can bind with core RNA polymerase (RNAP) and regulate gene transcription [20,21]. Recent reports have shown that RpoN is essential for biofilm detachment, flagellar formation, motility, and virulence of *V. alginolyticus* and other pathogens [22,23,24]. RpoN also affects the flagellar biogenesis-dependent motility in *Vibrio* spp. by regulating RpoN-dependent gene transcription [19]. Furthermore, the Class IV flagellar genes’ transcription of *Vibrio* spp. are regulated by RpoN-dependent activation of alternative sigma factors FliA (σ28) [25]. Another necessary sigma factor for flagellum regulation is the general stress response regulator RpoS (σ38), which is considered as the antagonist of RpoN and negatively controls flagellar genes’ expression and motility in bacteria [26,27,28].

*V. alginolyticus* is one of the major pathogens that leads to the *vibriosis* of aquatic animals and severe food safety concerns. However, except for its mediation of interbacterial competition [5,7], other roles of the *Vibrio alginolyticus* T6SS are still largely unclear. In this study, the impacts of *hcp2* on bacterial swarming motility, adhesive ability, and virulence against zebrafish were analyzed by constructing an *hcp2* mutant strain of *V. alginolyticus* HY9901. Furthermore, the proteomic and real-time fluorescence quantitative results confirmed the positive roles of *hcp2* on flagellar assembly. These data will be beneficial to further explore the pathogenicity of *V. alginolyticus.*

## 2. Materials and Methods

### 2.1. Bacterial Strains, Media, Plasmids, and Zebrafish Lines and Maintenance

The bacterial strains, plasmids, and zebrafish lines used in this study are shown in Supplementary Material Table S1. *Vibrio alginolyticus* strain HY9901 was kept in our laboratory [29]. *V. alginolyticus* was grown in tryptic soy broth (TSB, Huankai, Guangzhou, China) at 28 °C. *Escherichia coli* S17 strains were cultured in Luria broth (LB, Huankai, Guangzhou, China) at 37 °C. Adult AB zebrafish line purchased from China Zebrafish Resource Center (CZRC) was maintained under standard conditions [30]. Handling of zebrafish complied with “The Legislation of Guangdong Laboratory Animal Management Regulations (2010)”.

### 2.2. Construction and Physiology Analysis of the hcp2 Mutation

The primers for the construction of the *hcp2* mutation were designed based on the sequence of *hcp2* gene of *V. alginolyticus* strain HY9901 cloned by our laboratory (Supplementary Material Table S2). The PCR-amplified DNA fragment used for constructing the in-frame deletion mutation of *hcp2* was generated by overlap PCR [31]. Briefly, two PCR fragments were obtained from *V. alginolyticus* strain HY9901 genomic DNA with the primer pairs of *hcp2*-MF1/ *hcp2*-MR1 and *hcp2*-MF2 /*hcp2*-MR2 by using PrimeSTAR Max DNA Polymerase (TaKaRa, Kusatsu City, Japan). The resulting products generated a 707 bp fragment containing the DNA upstream of *hcp2* and a 650 bp fragment containing the DNA downstream of *hcp2*. A 20 bp overlap in the sequences permitted amplification of a 1357 bp product containing a deletion from nucleotides 58 to 447 of *hcp2* during a second PCR with primers of *hcp2*-MF1 and *hcp2*-MR2. The resulting product was linked into the suicide plasmid pLP12 by Exnase II recombinase (ClonExpress II, Vazyme, Nanjing, China) and the recombinant product was transformed into *E. coli* DH5α cultured with LB solid media containing 20 μg/mL chloramphenicol and 0.3% D-Glucose. The recombinant suicide plasmid pLP12-*hcp2* was confirmed by PCR with primers of pLP-UF/pLP-UR and subsequently transformed into *E. coli* β2163. *E. coli* β2163 containing the plasmid pLP12-*hcp2* was conjugated with wild-type *V. alginolyticus* strain HY9901 and co-incubated with LB plate containing 0.3 mM diaminopimelic acid (DAP) and 0.3% D-Glucose. Recipient cells were plated on LB supplemented with 0.3% D-Glucose and the antibiotics chloramphenicol to select the clone pLP12-*hcp2* that had integrated the vector by homologous recombination. Antibiotic-resistant colonies were isolated, identified with the primers of *hcp2*-TF/pLP-UTR, grown in LB to the late logarithmic phase, and plated on counter-selective LB containing 0.4% (*w*/*v*) L-arabinose. The selected colonies were confirmed by PCR using the primers of *hcp2*-TF/ *hcp2*-TR and direct sequencing. 

Growth ability and swarming motility assay were measured according to the method of Zhou et al. [32] and Moisi et al. [33], respectively, with a slight modification. Briefly, to measure the growth level of bacteria in TSB, overnight cultures of *V. alginolyticus* were adjusted to OD_600_ of 0.5 and subsequently inoculated into TSB with a dilution rate of 1:100 (*v*/*v*). Samples were removed every 1 h, and the optical density was measured at 600 nm. To measure swarming motility, the colony of *V. alginolyticus* was spotted on TSA plate containing 0.3% agar and 3% sodium chloride. The plate was analyzed after at least 12 h of incubation at 28 °C. 

### 2.3. Virulence Determination

The wild-type and *hcp2* mutation of *V. alginolyticus* were prepared as follows. Briefly, *V. alginolyticus* strains were cultured at 28 °C in TSB medium for 18 h. The overnight cultures were adjusted to OD600 of 0.5, subsequently inoculated into TSB with a dilution rate of 1:100 (*v*/*v*), and incubated at 28 °C with rotation until they reached the exponential phase. Then, the number of colonies were determined by flat colony counting method. For larval infection assay, 4 dpf (days post-fertilization) zebrafish were randomly transferred into 6-well plates. Each well contained 8~10 fish with 10 mL embryo medium, and the fish were immersed in 10^8^ CFU of *V. alginolyticus* strains; then, bacteria and fish were kept at 28 °C for 2~3 d. From 5 dpf, all the fish were fed by paramecium to maintain normal growth. The control larvae were raised normally without *V. alginolyticus* strains. For adult zebrafish challenged assay, 7-month-old fish were randomly transferred into 4 groups and were infected by intraperitoneal injection of 10^8^, 10^7^, 10^6^, and 10^5^ CFU of *V. alginolyticus* strains, respectively. As a control, the fish were injected with sterilized 10% (g/mL) NaCl. All the adult fish were kept at 28 °C for 12 hpi~14 hpi (hours post-injection), and mortality was recorded. Medium lethal dose (LD50) was used to estimate the bacterial virulence in zebrafish via LD50 data processing software 1.01 published by Blue Cosmos Studio.

### 2.4. Adhesion and Intestinal Colonization Assays

The overnight cultures were adjusted to OD600 of 0.5, subsequently inoculated into TSB with a dilution rate of 1:100 (*v*/*v*), and incubated at 28 °C with a rotation speed of 125 rpm until they reached the exponential phase. Plate colony counting method was used to determine bacterial concentration (CFU/mL). The cultures were centrifuged at 4000 rpm for 2 min, and the cells were resuspended with 5 ug/mL CellTracker™ CM-DiI dye (C7000, Invitrogen, Waltham, MA, USA) in 10% NaCl. Bacterial suspension was incubated at 37 °C for 5 min then at 4 °C for 15 min (low-temperature incubation helps to reduce bacterial endocytosis of dyes and facilitates the labeling of plasma membranes). After labelling was finished, bacteria were washed twice and resuspended in 10% NaCl. For adhesion experiment, 7~10 of 5 dpf transgenic zebrafish Tg (coro1a: eGFP) were co-incubated with 107 and 108 CFU of dye-marked bacteria at 28° for 42 hpi (hours post-immersion). Similarly, 5 dpf AB line zebrafish were challenged by 108 CFU dye-labelled *Vibrio alginolyticus* for 3 dpi (days post-immersion) and 5 dpi, respectively. Subsequently, the zebrafish were washed with PBS 3 times and imaged by a Nikon upright fluorescence microscope (Ni-E, Tokyo, Japan).

### 2.5. Construction of hcp2 Complementary Strain and Electron Microscopy Imaging

To create a complemental plasmid for the *hcp2* mutation, the *hcp2* gene was amplified by primers of com*hcp2*-F and com*hcp2*-R (Supplementary Material Table S2), and then the fragment obtained was cloned into PstI and XhoI sites of the pBBR1-MCS1 to construct pBBR1-MCS1-*hcp2*. The resulting plasmid was first transformed into *E. coli* DH5α and plasmid DNA was isolated. The correct pBBR1-MCS1-*hcp2* plasmid was transformed into *E. coli* S17-1 λpir. S17-1 λpir harboring the plasmid pBBR1-MCS1-*hcp2* was conjugated with *hcp2* mutation and co-grown with TSA overnight without antibiotics. The mixed culture was plated on TCBS with chloramphenicol, which only permitted vibrio spp. proliferation. The selected colonies were confirmed further by PCR analysis and sequencing. The constructing method of wild-type HY9901 and *hcp2* mutation harboring the empty vector pBBR1-MCS1 was the same as the complementary strain.

To observe flagellar morphology, *V. alginolyticus* strains of overnight cultures were negatively stained with 1% phosphotungstic acid (pH 7.4) on a Formvar carbon-coated grid and scanned by a transmission electron microscope (JEM-1400, Tokyo, Japan) [34]. Flagellar filament width was measured on electron micrographs recorded at a magnification of 30,000. For the phenotype observed, zebrafish were embedded with 1% (*m*/*v*) low-melting point agarose and imaged with a Nikon upright fluorescence microscope (Ni-E, Japan).

### 2.6. Proteomics Analysis of the Proteins Associated with Flagellum Assembly

#### 2.6.1. Protein Extraction, Digestion, TMT/iTRAQ Labeling, and HPLC Fractionation

For protein extraction, after overnight cultures were adjusted to OD600 of 0.5, wild-type HY9901 and Δ*hcp2* strains were inoculated into TSB with a dilution of 1:100 for 18 h of incubation, respectively, and then the cells were collected by centrifugation at 4000× *g* for 5 min at 4 °C. The sediments were sonicated in lysis buffer (8 M urea, 1% protease inhibitor cocktail). The remaining debris was removed by centrifugation at 12,000× *g* at 4 °C for 10 min. The supernatant was collected, and the protein concentration was determined with BCA kit according to the manufacturer’s instructions. For digestion, the protein solution was reduced with 5 mM dithiothreitol for 30 min at 56 °C and alkylated with 11 mM iodoacetamide for 15 min at room temperature in darkness. The protein sample was then diluted by adding 100 mM TEAB to urea concentration less than 2 M. Trypsin was added at 1:50 trypsin-to-protein mass ratio for the first digestion overnight and 1:100 trypsin-to-protein mass ratio for a second 4 h digestion. After trypsin digestion, the peptide was desalted by Strata X C18 SPE column (Phenomenex, Hong Kong, China) and vacuum dried. The peptide was reconstituted in 0.5 M TEAB and processed according to the manufacturer’s protocol for TMT kit/iTRAQ kit. The peptide mixtures were then incubated for 2 h at room temperature and pooled, desalted, and dried by vacuum centrifugation. For purification, the tryptic peptides were fractionated into fractions by high pH reverse-phase HPLC using Agilent 300Extend C18 column (5 μm particles, 4.6 mm ID, 250 mm length) (Santa Clara, CA, USA). Then, the peptides were combined into 18 fractions and dried by vacuum centrifuging. 

#### 2.6.2. LC-MS/MS Analysis

The tryptic peptides were dissolved in 0.1% formic acid (solvent A) and directly loaded onto a homemade reversed-phase analytical column (15 cm length, 75 μm i.d.). The gradient comprised an increase from 6% to 23% solvent B (0.1% formic acid in 98% acetonitrile) over 26 min, 23% to 35% in 8 min, rose to 80% in 3 min, and then was maintained at 80% for the last 3 min. All this occurred at a constant flow rate of 400 nL/min on an EASY-nLC 1000 UPLC system. The peptides were subjected to NSI source followed by tandem mass spectrometry (MS/MS) in Q Exactive^TM^ Plus (Thermo, Waltham, MA, USA) coupled online to the UPLC. The electrospray voltage applied was 2.0 kV. The *m*/*z* scan range was 350 to 1800 for the full scan, and intact peptides were detected in the Orbitrap at a resolution of 70,000. Peptides were then selected for MS/MS using NCE setting of 28 and the fragments were detected in the Orbitrap at a resolution of 17,500. A data-dependent procedure that alternated between one MS scan followed by 20 MS/MS scans with 15.0 s dynamic exclusion was carried out. Automatic gain control (AGC) was set at 5 × 10^4^. The fixed first mass was set as 100 *m*/*z*.

#### 2.6.3. Volcano Plot Analysis

To analyze the differential expression of flagellar proteins, a volcano plot was generated based on the proteome data. The threshold of fold change (FC) ratio and *p*-value were set as above 1.5 and less than 0.05, respectively, which were considered as statistically different. The flagellum protein level between wild-type HY9901 and Δ*hcp2* was quantified by the LFQ intensity value from proteome data.

### 2.7. Real-Time Quantitative PCR (RT-qPCR)

For bacteria, adjusted to OD600 of 0.5 in overnight cultures, *V. alginolyticus* strain HY9901 harboring the empty vector PBBR1-MCS1 (HY9901-ev), HY9901 Δ*hcp2* strain harboring the empty vector (Δ*hcp2*-ev), and Δ*hcp2* complementary strain (Δ*hcp2*-com), respectively, were inoculated on TSB with a dilution of 1:100 and incubated at 28 °C with agitation for 18 h. Total RNA was extracted and purified using RNAprep Pure Cell/Bacteria Kit (TIANGEN, Beijing, China) according to the manufacturer’s instructions. Reverse transcription PCR was conducted by using a PrimeScript™ RT reagent Kit (Takara, Kusatsu City, Japan) with 500 ng of total RNA in each reaction according to the manufacturer’s instructions. RT-qPCR was performed using LightCycler 480 detection system (Roche, Penzberg, Germany) with a PerfectStart^®^ Green qPCR SuperMix (+Dye I) (TransGen, Beijing, China). 

Zebrafish were challenged with *V. alginolyticus* for 43 h at the beginning of 4 dpf; then, the larvae were collected and washed with PBS thrice. Total zebrafish RNA was extracted using Trizol regent (Invitrogen, Waltham, MA, USA) and then was reverse transcribed into cDNA with PrimeScript RT Reagent Kit (TaKaRa, Kusatsu City, Japan) according to the manufacturer’s instructions. RT-qPCR was performed using a LightCycler 480 detection system (Roche, Penzberg, Germany) with a PerfectStart^®^ Green qPCR SuperMix (+Dye I) (TransGen, Beijing, China). All the sequences of RT-qPCR primers are listed in Supplementary Material Table S2.

### 2.8. Statistical Analysis

The results are presented as means ± standard error (mean ± SEM). All the experiments were conducted at least 3 times. Two-tailed Student’s *t*-test (for two groups) or one-way analysis of variance (ANOVA followed by Tukey’s test for three or more groups) were applied to determine statistical significance with Prism 5 software (GraphPad Software). Confidence intervals of 95% for data analyses were acquired for all experiments. *p*-value less than 0.05 (*p* < 0.05) was considered significant. 

## 3. Results

### 3.1. Loss of hcp2 Reduced the Swarming Motility of Vibrio alginolyticus

**Figure 1:**
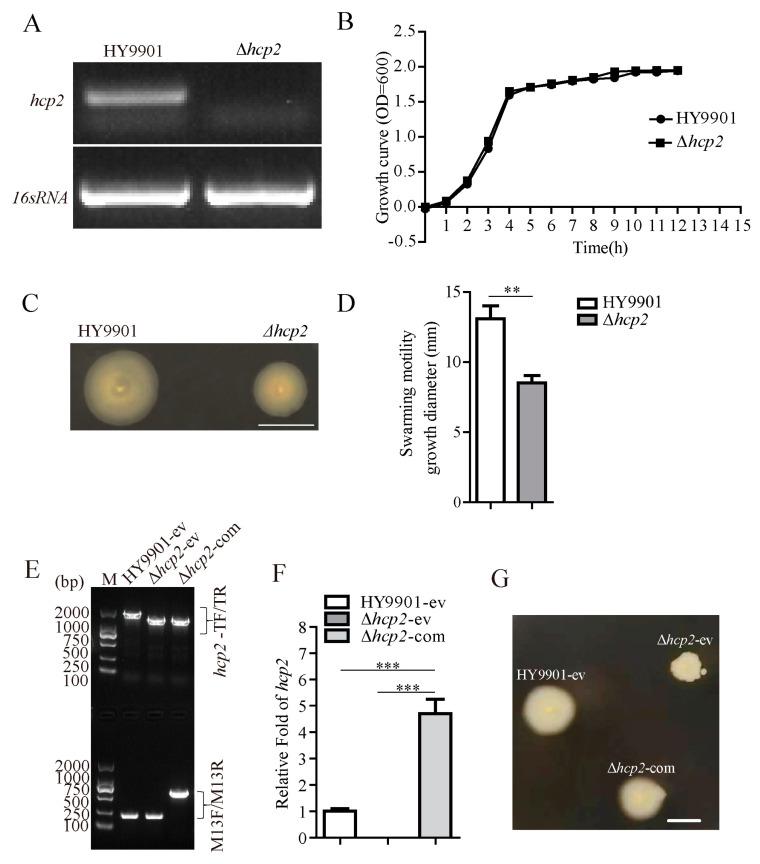
Establishment and physiological analysis of *hcp2* mutation. (**A**) Validation of *hcp2* mRNA level in *hcp2* mutant strain and wild-type HY9901 by RT-PCR. (**B**) Growth curve of wild-type HY9901 and Δ*hcp2* strain. (**C**) Observation of swarming motility in wild-type HY9901 and Δ*hcp2* strain. (**D**) Measurement results of motility growth diameter in wild-type HY9901 and Δ*hcp2* strain. (**E**) The result using PCR verifying *hcp2* complementary strain of *V. alginolyticus*. (**F**) The verification of *hcp2* transcription level after recombinant plasmid transformed into *Vibrio alginolyticus* by RT-qPCR. (**G**) Validation of swarming motility in wild-type, *hcp2* mutation, and complementary strains. M: DL2000 DNA Marker. HY9901-ev: the wild-type strain harboring empty vector pBBR1-MCS1; Δ*hcp2*-ev: the Δ*hcp2* strain harboring empty vector pBBR1-MCS1; Δ*hcp2*-com: the Δ*hcp2* strain harboring complementary vector pBBR1-MCS1-*hcp2*; *hcp2*-TF/TR: the genotyping result of genomic *hcp2* using the *hcp2*-TF/TR primers; M13F/13R: the genotyping result of plasmid using the M13F/13R primers; Scale bar: 10 mm in (**C**,**G**); *** *p* < 0.001, ** *p* < 0.01.

Hcp, a hallmark component of the T6SS, was initially identified as a secreted protein of *Vibrio cholerae* [35]. The current study revealed that Hcp also functions as an exported effector and chaperone of Type VI secretion substrates in Gram-negative bacteria [2,10,36]. Hcp has been proven to be involved in bacterial motility, adherence, and cytotoxicity to host cells by regulating the flagellar system in various pathogens [12,13,15,16]. However, for *Vibrio alginolyticus*, the roles of Hcp on bacterial motility remains unknow. Using the suicide plasmid-mediated homologous recombination, a *hcp2* deletion mutation (Δ*hcp2*) was constructed. Using a housekeeping gene, 16s RNA, as control for normalization, the RT-PCR result confirmed that the *hcp2* transcription level of the mutation was clearly decreased compared to HY9901 wild-type (Figure 1A). The mutant showed a similar growth level to the wild-type strain cultured in TSB (Figure 1B). Meanwhile, notable impairment of the swarming motility was observed in the Δ*hcp2* of *V. alginolyticus* compared with wild-type HY9901 (Figure 1C,D; *p* < 0.05). To further clarify the effect of *hcp2* on motility, a Δ*hcp2* complementary strain was constructed and confirmed by RT-qPCR (Figure 1E,F). As expected, supplementation with *hcp2* partially restored the swarming ability in the Δ*hcp2* strain (Figure 1G).

### 3.2. Attenuated Pathogenicity of Δhcp2 Strain against Zebrafish

**Figure 2:**
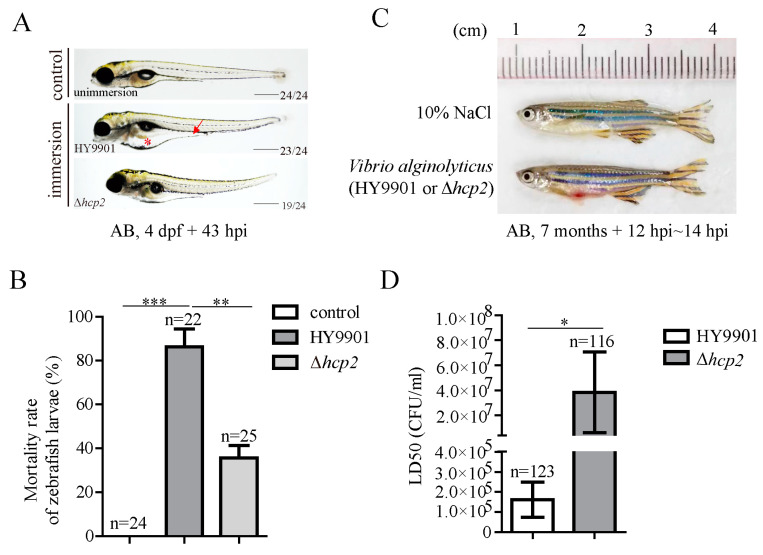
Comparison of virulence on zebrafish between wild-type HY9901 and *hcp2* mutation. (**A**) Zebrafish phenotype after *Vibrio alginolyticus* immersion for 43 hpi at 4 dpf larvae. (**B**) Mortality analysis of AB zebrafish larvae after 69 h treatment in uninfected, wild-type HY9901, and Δ*hcp2* strain groups, respectively. (**C**) Adult zebrafish phenotype after intraperitoneal injection by wild-type HY9901 or Δ*hcp2* strain. (**D**) Comparison of *Vibrio alginolyticus* LD50 against adult zebrafish; red arrow: gut edema; red asterisk: belly edema. CFU: colony forming unit; dpf: days post-fertilization; hpi: hours post-immersion. The bottom right value in Figure 2A represents the phenotype ratio; *n*: the number of zebrafish analyzed; *** *p* < 0.001, ** *p* < 0.01, * *p* < 0.05; scale bars: 500 μm in Figure 2A.

As mentioned above, Hcp family proteins have been proved to participate in modulating pathogenicity in many pathogens. To compare the virulent differences between the wild-type strain HY9901 and the Δ*hcp2* strain, larval and adult zebrafish were challenged with *V. alginolyticus*. After 43 hpi (hours post-immersion) of 4 dpf AB larvae, more severe edema in the zebrafish larval belly (red asterisk) and gut (red arrow) were observed compared to after the zebrafish were challenged by the wild-type strain then immersed in Δ*hcp2* counterparts (Figure 2A). And then, around 69 hpi, those larvae infected by the Δ*hcp2* strain had a lower mortality rate compared to those fish challenged by wild-type bacteria (Figure 2B). Consistently, a red and swollen phenotype on the belly was observed in adult zebrafish injected with *V. alginolyticus*, and IC50 value was significantly increased in the Δ*hcp2* infection group (Figure 2C,D). 

### 3.3. Reduced Adhesive Ability of Δhcp2 Strain on the Zebrafish larval Surface and Intestinal Tract

**Figure 3:**
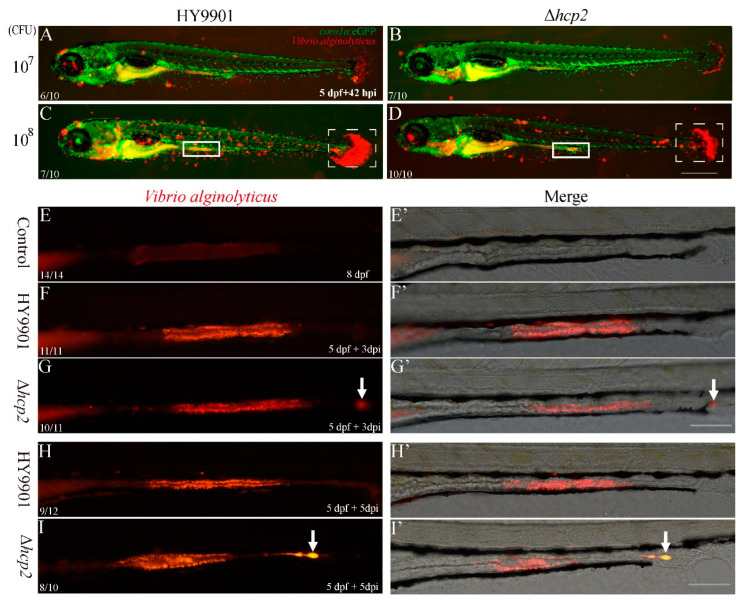
Adhesive and intestinal colonization capacity of *Vibrio alginolyticus* against zebrafish. (**A**–**D**) Comparison of adhesion between wild-type HY9901 and *hcp2* mutant strains on zebrafish larval surface at 5 dpf after 42 hpi. (**E**–**I’**) Comparison of colonization ability between wild-type HY9901 and *hcp2* mutant strains on zebrafish intestinal canal at 5 dpf after 3 dpi and 5 dpi, respectively. Red font: DiI dye-labelled *Vibrio alginolyticus*; green font: green fluorescent protein-labelled zebrafish macrophage; white rectangle: zebrafish intestine colonized by *Vibrio alginolyticus*; white dotted rectangle: zebrafish caudal fin; white arrow: *Vibrio alginolyticus* outside of zebrafish anus; CFU: colony forming unit; n/n: number of fish with phenotype/number of total imaged fish; dpf: days post-fertilization; hpi or dpi: hours (days) post-immersion; scale bar: 500 μm in (**D**), 100 μm in (**G**,**I’**).

Motility is a fundamental function of bacteria and is responsible for the initial colonization to the host. Before that, adhesive capacity on the host is the first event in bacterial infection. To clarify that the attenuated virulence of the Δ*hcp2* strain was partially related to reduced adhesion, a comparison of adhesive capacity on zebrafish was performed between Δ*hcp2* and HY9901 wild-type strains. We found a clear decrease of red dye-labelled *Vibrio alginolyticus* of the Δ*hcp2* strain on the whole zebrafish trunk surface and tail at 5 dpf after 42 hpi treatment (Figure 3A vs. Figure 3B, Figure 3C vs. Figure 3D). Interestingly, the dye-labelled strains seemed to be surrounded by GFP-labelled macrophage in the zebrafish intestinal canal (white rectangle in Figure 3), which indicated that the phenotype of edema from zebrafish abdomen and gut displayed in Figure 2 was indeed caused by *Vibrio alginolyticus* infection. To further confirm whether the Δ*hcp2* strain was more easily excreted from the intestinal canal of the zebrafish as its motility was impaired, 5 dpf AB larvae were infected with dye-labelled *Vibrio alginolyticus*. Consistent with what we observed in a transgenic zebrafish assay, the *hcp2* mutant bacteria were weakened in colonization (Figure 3E–I’) and easily excreted to the outside of zebrafish canal by the intestinal movement (white arrow in Figure 3).

### 3.4. A Malformed Morphology of Flagellar Filaments in hcp2 Mutation of Vibrio alginolyticus

**Figure 4:**
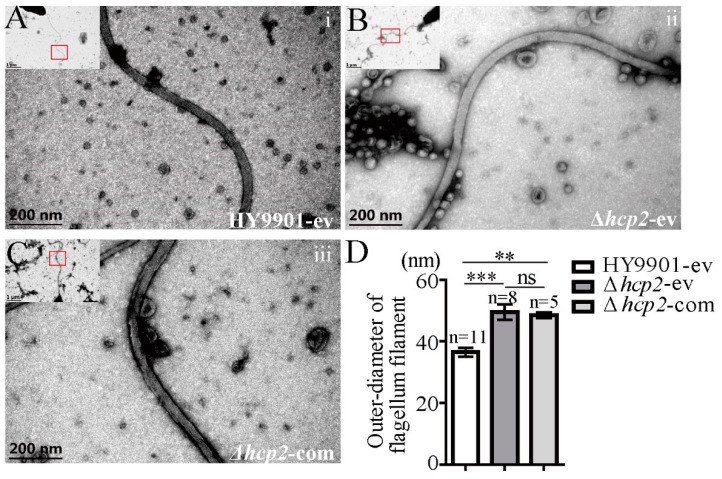
Imaging of the flagellar filament morphology by electron microscopy. (**A**–**C**) Negative-staining EM images of the sheathed polar flagella of wild-type HY9901, Δ*hcp2* strain, and Δ*hcp2* complementary strain; (**i**–**iii**) are high-magnification scans of the red line-marked regions in each top-left image, respectively. (**D**) Measurement of outer-diameter of flagellar filaments in wild-type HY9901, Δ*hcp2* strain, and Δ*hcp2-*complementary strain. HY9901-ev: the wild-type strain harboring empty vector pBBR1-MCS1; Δ*hcp2*-ev: the Δ*hcp2* strain harboring empty vector pBBR1-MCS1; Δ*hcp2*-com: the Δ*hcp2* strain harboring complementary vector pBBR1-MCS1-*hcp2*; *n*: the number of analyzed bacteria at 30,000× magnification. *** *p* < 0.001, ** *p* < 0.01; ns: no significance.

The bacterial polar flagellum is essential for mediating motility, adhesion, and pathogenicity. To further verify whether *hcp2* influenced the flagellar morphology and resulted in the disablement of motility, negative-staining TEM scanning was performed. As shown in Figure 4B, a severely hollow-like structure of flagellar filament was observable in the Δ*hcp2*-ev strain compared with the HY9901-ev strain (Figure 4A), and this morphology of filament could be rescued partially by the complement of *hcp2* in the mutant strain (Figure 4C). Then, the outer diameter of each flagellar filament was measured based on the TEM data. The result showed that the outer diameter of filament of the Δ*hcp2*-ev mutation was significantly enlarged (as shown in Figure 4D, the mean ± standard error of filament width in each of the strain Δ*hcp2*-ev was 49.5 ± 2.5 nm vs. 36.48 ± 1.5 nm in wild-type HY9901-ev; *p* < 0.001). Nevertheless, there was no difference in the outer diameter of filament between the Δ*hcp2*-ev and the Δ*hcp2*-com strain (mean ± standard error of the filament width in Δ*hcp2*-ev bacteria were 49.5 ± 2.5 nm vs. 48.51 ± 0.66 nm in the Δ*hcp2*-com strain; *p* > 0.05). 

### 3.5. Expression and Transcription Levels Analysis of Flagellin and Flagellum Export Apparatus by Quantitative Proteomics and RT-qPCR, Respectively

**Figure 5:**
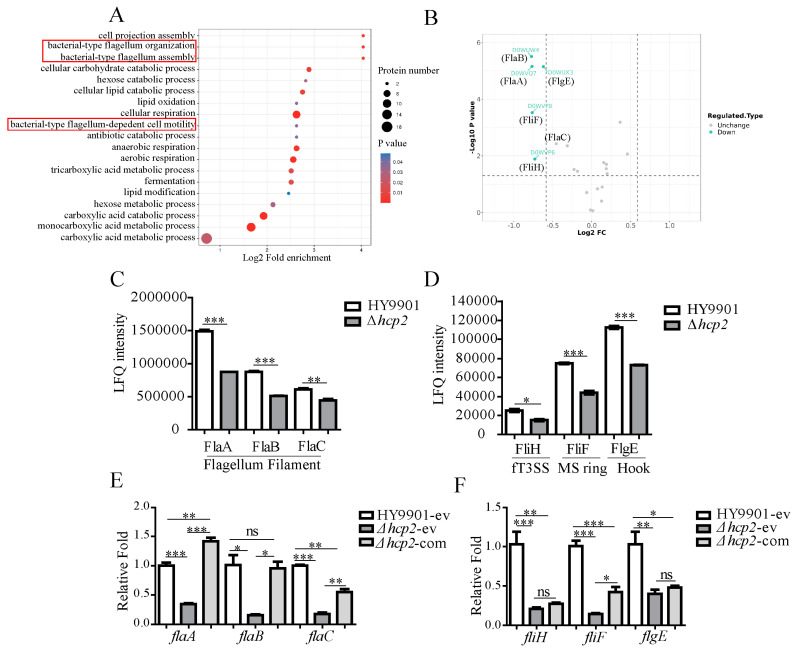
Flagellin and flagellum export apparatus-associated genes’ expression of *V. alginolyticus* by quantitative proteomics and RT-qPCR analysis. (**A**) The enrichment result of bacterial motility and flagellum assembly-associated biological process (red rectangle) from all differential expression proteins in Δ*hcp2* strain compared with wild-type HY9901. (**B**) Volcano graphs for the analysis of the polar flagellum assembly-associated proteins based on proteomics data and differential expression proteins between Δ*hcp2* strain and wild-type HY9901. (**C**) Protein quantitative result of flagellins FlaA, FlaB, and FlaC in the wild-type HY9901 and the Δ*hcp2* strain by LFQ intensity. (**D**) Protein quantitative result of FliH, FliF, and FlgE in the wild-type HY9901 and the Δ*hcp2* strain by LFQ intensity. (**E**) The influence on transcriptional levels of flagellin genes *flaA*, *flaB*, and *flaC* by *hcp2* using RT-qPCR analysis. (**F**) The influence on transcriptional levels of *fliH*, *fliF*, and *flgE* by *hcp2* using RT-qPCR analysis. HY9901-ev or Δ*hcp2*-ev: the wild-type strain or *hcp2* mutant strain harboring empty vector; Δ*hcp2-*com: the mutant strain harboring *hcp2* complementary plasmid. *** *p* < 0.001, ** *p* < 0.01, * *p* < 0.05; ns: no significance.

Emerging studies have demonstrated the potential regulatory role of the T6SS on the bacterial flagella system [37]. A study of *Citrobacter freundii* [15], *Erwinia amylovora* [38]*, Pseudomonas genus* [39], and *Vibrionaceae* [40] recorded the involvement of the T6SS in modulating flagellar gene expression and secretion. Consider that the normal assembly of polar flagella is crucial for motile flagellated bacteria [41]. Thus, the effect of *hcp2* on bacterial flagellar assembly was assessed. Our previous studies confirmed the flagellin of *V. alginolyticus* HY9901 are composed of three flagellum genes encoded FlaA, FlaB, and FlaC [42,43]. Moreover, flagellin from *V. alginolyticus* HY9901 has been proven to be one of the important factors for bacterial intestinal colonization [44]. By proteomic analysis, the differential expression proteins were significantly enriched in the processes of bacterial-type flagellum organization, assembly, and flagellum-dependent cell motility (red rectangle in Figure 5A). All the identified proteins associated with flagellar assembly were analyzed by volcano plot, in which the differential proteins were marked (Figure 5B). The intracellular protein levels of flagellin FlaA, FlaB, and FlaC were remarkably decreased in the Δ*hcp2* strain (Figure 5B,C). Furthermore, the expression levels of flagellar type III secretion system (fT3SS)-associated protein FliH, MS ring protein FliF, and flagellum hook protein FlgE were also decreased in the mutant strain (Figure 5D). In RT-qPCR assay, the transcriptional levels of *flaA*, *flaB*, and *flaC* genes were significantly reduced in the Δ*hcp2* strain and were partially restored by supplying *hcp2* (Figure 5E). 

### 3.6. The Transcriptions of Sigma Factors rpoN and fliA Controlling Flagellin Biosynthesis and Export Were Positively Influenced by hcp2 in Vibrio alginolyticus

**Figure 6:**
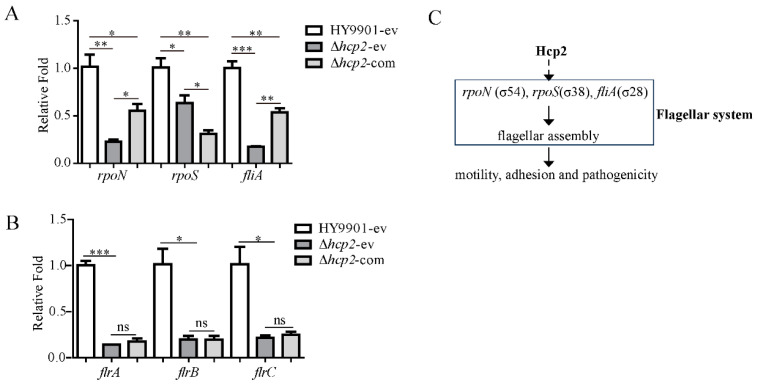
The modulatory role of *V. alginolyticus* Hcp2 protein on the flagellar system and pathogenicity. (**A**) Transcription levels analysis of sigma factors *rpoN*, *rpoS*, and *fliA* in the wild-type, *hcp2* mutation, and complementary strain by RT-qPCR. (**B**) Transcription level analysis of master regulon *flrA* and its downstream genes *flrB* and *flrC* in the wild-type, *hcp2* mutation, and complementary strain by RT-qPCR. (**C**) General view of the roles of Hcp2 protein on flagellar system and pathogenicity of *V. alginolyticus*. Black dotted arrow: positive impact on flagellar assembly through Hcp2 protein; *** *p* < 0.001, ** *p* < 0.01, * *p* < 0.05; ns: no significance.

In bacteria, sigma factors RpoN, RpoS, and FliA control flagellar gene transcriptional activation [41,45], but RpoS and RpoN are reciprocal function factors for controlling flagellar gene expression in *Escherichia coli* and *Edwardsiella tarda* [27,46,47]. FlrA is another important master regulon that initiates flagellar gene expression in an RpoN-dependent manner, which is required for proper flagellation and includes the polar flagellar number and morphology related to bacterial motility [48]. Additionally, another study found that flagellin mutants or *flrA*-, *flrB*-, or *flrC*-silenced strains affect filament morphology and the swimming, adhesion, and biofilm formation ability for its pathogenesis in *Stenotrophomonas maltophilia* and *Vibrio alginolyticus* [49,50]. Hence, we investigated whether the transcriptional levels of these flagellar regulons were affected by *hcp2*. The RT-qPCR result showed that *hcp2* deletion resulted in transcriptional reduction of sigma factors *rpoN*, *fliA*, and *rpoS*. Their mRNA levels were partially restored in the Δ*hcp2* complementary strain (Figure 6A). Meanwhile, there was no significant transcriptional restoration of the regulators *flrA*, *flrB*, and *flrC in* the complementary strain, though their mRNA levels were reduced in *hcp2* mutation (Figure 6B), which indicated that the synthesis and export of flagellin FlaA, FlaB, FlaC were mainly modulated by *rpoN* and *fliA*. This finding was consistent with previous reports. Taken together, the results suggested that *hcp2* played positive and vital roles on bacterial motility, adhesive ability, and pathogenicity mainly through affecting the transcription of sigma factors in *Vibrio alginolyticus* HY9901 (Figure 6C).

## 4. Conclusions

As the hallmark component of the T6SS, hemolysin-coregulated protein (Hcp) is required for functional T6SS, which has a regulatory role in flagellar assembly and bacterial pathogenesis in Gram-negative bacteria. In this study, the roles of *hcp2* in *Vibrio alginolyticus* HY9901 were investigated by constructing a mutant strain with an in-frame deletion of the *hcp2* gene. The results suggested that *Vibrio alginolyticus* Hcp2 protein was involved in bacterial motility ability, adhesive capacity, and virulence through affecting the expression levels of flagellin and flagellar export proteins. Further RT-qPCR results indicated that *hcp2* had a positive influence on the transcriptional levels of flagellar assembly-associated sigma factors. *flrA* transcription was not clearly restored in the Δ*hcp2* complementary strain, suggestion that the effect of Hcp2 on the motility was independent of the FlrA-RpoN pathway and implying the possible involvement of other regulatory mechanisms in *flrA* expression in *V. alginolyticus*. For example, the two-component system, a crucial bacterial signal transduction system for modulating gene expression (in genes such as *flrA*, *flrB*, and *flrC*) to maintain the balance in the amount of regulator input and output via feedback mechanisms, may be involved [51]. A previous study has shown a novel role for Hcp which directly interacts with the RpoN-dependent T6SS regulator VasH to quickly adjust T6SS expression in several pathogens [52]. Therefore, there might be unrecognized Hcp2–protein interaction for mediating flagellar regulons’ expression in *Vibrio alginolyticus*. This study strengthened the evidence for the regulatory role of the T6SS on flagellar assembly and expanded our understanding of the pathogenesis of *Vibrio alginolyticus*.

## Data Availability

All data in this study are available from the corresponding author upon reasonable request.

## Supplementary Materials

The following supporting information can be downloaded at: https://www.mdpi.com/article/10.3390/microorganisms11122893/s1. Table S1: Plasmids and bacterial strains used in this study; Table S2: Sequences of primers used in this study.
