# Supplementary material for: Roles of Hcp2, a Hallmark of T6SS2 in Motility, Adhesive Capacity, and Pathogenicity of Vibrio alginolyticus
Source: Microorganisms. 2023 Nov 30;11(12):2893. doi: 10.3390/microorganisms11122893 (PMC10745990; doi:10.3390/microorganisms11122893)
Supplement: Supplementary file 1 [file microorganisms-11-02893-s001.zip › Table S1.pdf]

Table S1. Plasmids and bacterial strains used in this study

Strains and plasmids	Genotype and relevant characteristics	Source or references
E. coli strains		
DH5α	F- φ80d *lac*ZΔM15 Δ(*lac*ZYA-*arg*F) U169 *rec*A1 *end* A1 *hsd*R17(rk-, mk+) phoA, *sup*E44λ- *thi*-1 *gyr*A96 *rel*A1 *pho*A	TransGen Biotech
β2163	F–, RP4-2-Tc::Mu ΔdapA::(erm-pir)	Demarre et al., 2005
S17-1 λpir	Tpr Smr recA thi pro hsdR−M+RP4:2-Tc: Mu:Km Tn7 λpir	Simon et al., 1983
S17-1-pBBR1-MCS1-*hcp2*	S17-1 containing plasmid of pBBR1-MCS1-*hcp2*, Cmr	This study
Vibrio aginolyticus		
HY9901	Wild-type, isolated from the diseased fish	Cai et al., 2007
Δ*hcp2*	HY9901 carrying an in-frame deletion of *hcp2*	This study
HY9901-pBBR1-MCS1	HY9901 containing plasmid of pBBR1-MCS1, Cmr (HY9901-ev)	This study
Δ*hcp2*-pBBR1-MCS1	HY9901Δ*hcp2* containing plasmid of pBBR1-MCS1, Cmr (Δ*hcp2*-ev)	This study
Δ*hcp2*-pBBR1-MCS1-*hcp2*	HY9901Δ*hcp2* containing plasmid of pBBR1-MCS1-*hcp2*, Cmr (Δ*hcp2*-com)	This study
Plasmids		
pMD19-T	Cloning vector; Ampr	TaKaRa
pBBR1-MCS1	Expression vector; pBBR1 Rep, pBBR1 oriV; Cmr	Kovach et al., 1995
pLP12	oriTRP4 oriVR6Kvmi480 PBAD, Cmr	Luo et al., 2015
pLP12-hcp2	pLP12 derivative containing homologousarms of hcp2	This study
