# Supplementary material for: Roles of Hcp2, a Hallmark of T6SS2 in Motility, Adhesive Capacity, and Pathogenicity of Vibrio alginolyticus
Source: Microorganisms. 2023 Nov 30;11(12):2893. doi: 10.3390/microorganisms11122893 (PMC10745990; doi:10.3390/microorganisms11122893)
Supplement: Supplementary file 1 [file microorganisms-11-02893-s001.zip › Table S2.pdf]

Table S2. Sequences of primers used in this study

Primer	Sequence (5′−3′)	Targeting site	Source or references
hcp2-MF1	GGAATCTAGACCTTGAGTCGGAACA CAGGGACCGAAACGACGATG	Upstream of *hcp2*	This study
Hcp2-MR1	GACCTTTAACACCGCTGTAGCCCTC GGCTGTTGCCTCACCCT		
hcp2-MF2	AGGGTGAGGCAACAGCCGAGGGCT ACAGCGGTGTTAAAGGTC	Downstream of *hcp2*	This study
hcp2-MR2	ACAGCTAGCGACGATATGTCGCGTA TGGGCAGACTTAGTATGTG		
pLP-UF	GACACAGTTGTAACTGGTCCA	Recombinant plasmid pLP12 with *hcp2* fusion fragment	This study
pLP-UR	CAGGAACACTTAACGGCTGAC		This study
hcp2-TF	CACTATTATTATTATGACCGC	Genomic *hcp2*	This study
hcp2-TR	GTCAGAATTTGCGTTTCATGCC		
com*hcp2*-F	TGCActgcagATGCAGTCTAATACGTAT CTT (pstI)	*hcp2* cds	This study
com*hcp2*-R	CCGctcgagTTACATTTGTTGACCTTTA AC (xhoI)		
RT-qPCR			
hcp2-qF	TCTGTAGATTGGAGTGTAGGT	*hcp2* cds	This study
hcp2-qR	AACGGTCTTGAAGTTTAGTG		
flaA-qF	AGCGTTTGTCTTCGGGTTA	*flaA* cds	This study
flaA-qR	GCTACACGTTCTGCTTTTG		
flaB-qF	CGTTTGTCTTCAGGTTTT	*flaB* cds	This study
flaB-qR	GTTGCGAACTGCTACATC		
flaC-qF	ACTTCATTTGGTGGTCGT	*flaC* cds	This study
flaC-qR	AACATTGCCGTCTTCGTC		
flgE-qF	ACGACGAGTTCTTGTTAGGTT	*flgE* cds	This study
flgE-qR	AGAGGTTGAACGGTTGTATGT		
fliH-qF	TTATCAAGAAGGCTTACATC	*fliH* cds	This study
fliH-qR	AGCATTCATCAACTCCAA		
fliF-qF	CAAGAAGCGTCTGCGTCGGTAT	*fliF* cds	This study
fliF-qR	CTGAGCTGAGTAAGCGGCCATG		
rpoN-qF	AGGCAACCGTGACTACAA	*rpoN* cds	This study
rpoN-qR	AGGAGTGATGCGACTACC		
rpoS-qF	TCTCACCTTTACTTACTGCC	*rpoS* cds	This study
rpoS-qR	CTCGCTCTATCGTTTGTC		
fliA-qF	GAAGTAGCCACGCATCTC	*fliA* cds	This study
fliA-qR	CTGTCATCGGTTTCATCC		
flrA-qF	CGTTTTCCTTTGCCTGTT	*flrA* cds	This study
flrA-qR	TTTGCTTCCGTGTTTGAT		

flrB-qF	ATCCAGGCGGTGTTTGAC	*flrB* cds	This study
flrB-qR	CAGCGGCGTTCTTACTTG		
flrC-qF	CGACCGTTGCGTTAGAAA	*flrC* cds	This study
flrC-qR	TACTGGGCGGCTGACACT		
16SrRNA-F	TTGCGAGAGTGAGCGAATCC	*16SrRNA* cds	This study
16SrRNA-R	ATGGTGTGACGGGCGGTGTG		
